# Dataset of academic performance evolution for engineering students

**DOI:** 10.1016/j.dib.2020.105537

**Published:** 2020-04-18

**Authors:** Enrique Delahoz-Dominguez, Rohemi Zuluaga, Tomas Fontalvo-Herrera

**Affiliations:** a Universidad Tecnológica de Bolívar, Colombia; b Universidad de Cartagena, Colombia

**Keywords:** Learning analytics, Educational data mining, Predictive evaluation, Continuous assessment, Education

## Abstract

This data article presents data on the results in national assessments for secondary and university education in engineering students. The data contains academic, social, economic information for 12,411 students. The data were obtained by orderly crossing the databases of the Colombian Institute for the Evaluation of Education (ICFES). The structure of the data allows us to observe the influence of social variables and the evolution of students' learning skills. In addition to serving as input to develop analysis of academic efficiency, student recommendation systems and educational data mining. The data is presented in comma separated value format. Data can be easily accessed through the Mendeley Data Repository (https://data.mendeley.com/datasets/83tcx8psxv/1).

Specifications table

**Table:** 

Subject	Social Sciences
Specific subject area	Education
Type of data	Table (Data Frame)
How data were acquired	Collection, adaptation and adjustment of information from the evaluations carried out by the Colombian Institute for the Evaluation of Education (ICFES)
Data format	Raw, analyzed and descriptive statistical data
Parameters for data collection	The data collection process was done under the rational analysis of the researchers, identifying the criteria that could be useful for analyzing the academic performance of Engineering students in two periods. First, the evaluation made at the end of high school and Second the evaluation carried out at the end of their professional training. For this, database cross-section criteria were used that allowed the association of the information of the secondary education stage with the professional training in Engineering.
Description of data collection	The observations correspond to the results of the evaluation in two moments of education for Engineering students in Colombia. The first moment corresponds to the results of the secondary evaluation and the second moment to the results of the professional evaluation, in addition variables of the social context in which the students live are added
Data source location	Bogotá, Colombia
Data accessibility	The data is available at https://data.mendeley.com/datasets/83tcx8psxv/1

## Value of the data

• The data shown are very useful for the development of tools to control the direction of educational processes, particularly at the levels of secondary and professional education. This is possible because the configuration of the data set allows analyzing the relative contribution of the variables, in addition to the influence that one variable has on others, for example, the influence that the university or college has on the final score
• The scores of student evaluations are useful for performing efficiency analyses, considering both High Schools and universities as Decision Making Units (DMU's)
• The variables present in the dataset are fit to create prediction, classification and evaluation models of academic and social variables.
• Social variables such as socioeconomic status are useful to understand their influence on the results of their tests; on the other hand, the gender distribution variable by career could be used to analyze the situation of women in Engineering from Colombia.
• It doesn't exist a public common student ID that enables merges the databases. For that purpose, a formal request was presented to the Colombian Institute for Assessment of Quality Education, to indicate the linking ID for each student's records for both High School and University scores on National standardized tests. Besides a conscious process of cleaning and debugging was performed to guarantee the anonymous of the records.

## 1 Data description

The data set contains 12,411 observations where each represents a student and has 44 variables. The variables correspond to the student's personal information (categorical) and the result obtained in the assessments (numerical). The academic assessment is recorded at two moments of the student life. First, the scores of the national standardized test at the final year of the high school (Saber 11), evaluating five generic academic competencies. Mathematics (MAT_S11), assesses the skills of students to face situations that may be resolved with the use of some math tools. Critical Reading (CR_11), Assesses the skills needed to understand, interpret and evaluate texts that can be found in everyday life and at academic non-specialized contexts. Citizen Competencies (CC_S11), assesses the student's knowledge and skills that allow him to understand the social world from the perspective of social sciences and place this understanding as a reference in the exercise of his role as a citizen. Biology (BIO_S11), assesses the ability of the student to explain how some phenomena of nature occur based on observations, patterns and concepts of scientific knowledge. English (ENG_S11), assesses the competence to communicate effectively in English.

The second moment of academic assessment is at the final year of the professional career on Engineering, recorded on the national standardized test for higher education (SABER PRO). Similar to SABER 11 test, five generic academic competencies are assessed. Critical Reading (CR_SPRO), assesses the ability to understand a text either locally or globally and the critical approach to it. Quantitative reasoning (CR_PRO), assesses the ability to understand and manipulate quantitative data in different representations whether tables, graphs or diagrams. Citizen competencies (CC_PRO), assesses the concept of citizenship and inclusive coexistence within the framework proposed by the Colombian constitution. Written communication (WC_PRO), assesses student's ability to transmit in writing his ideas related to a topic. English (ENG_PRO), assesses the competence to communicate effectively using the English language.

The information corresponding to students personal information level was fulfilled by the student at the enrolment to the exam. For example, the variable socioeconomic level in Colombian is related to the Neighbourhood where the student lives. The variable ‘sisben’ refers to the economic aid program that the Colombian government grants to low-income families to improve their quality of life. The variables Internet, TV, Computer, WASHING_MCH, MIC_OVEN, CAR, DVD, FRESH, PHONE and MOBILE, indicate if in the student's home there are said services or appliances, with answer categories Yes / No.

The data can be accessed in the Mendeley data repository and downloaded in xlsx spreadsheet format. The data dimension is 12,411 rows, each corresponding to a student and 44 variables.

The gender distribution of students corresponds to 5043 (40.63%) for women and 7368 (59.37%) for men. To better illustrate the dataset, Tables 1 and 2 are presented for their description.

**Table 1:** Description of numeric variables.

Variable	Full name	Mean	Standard Deviation	Max	Min
MAT_S11	Mathematics	64.32	11.87	100	26
CR_S11	Critical Reading	60.78	10.03	100	24
CC_S11	Citizen Competencies S11	60.71	10.12	100	0
BIO_S11	Biology	63.95	11.16	100	11
ENG_S11	English	61.80	14.30	100	26
QR_PRO	Quantitative Reasoning	77.42	22.67	100	1
CR_PRO	Critical Reading	62.20	27.67	100	1
CC_PRO	Citizen Competencies SPRO	59.19	28.99	100	1
ENG_PRO	English	67.50	25.49	100	1
WC_PRO	Written Communication	53.70	30.00	100	0
FEP_PRO	Formulation of Engineering Projects	145.48	40.12	300	1
G_SC	Global Score	162.71	23.11	247	37
PERCENTILE	Percentile	68.45	25.87	100	1
2ND_DECILE	Second Decile	3.89	1.25	5	1
QUARTILE	Quartile	3.19	0.98	4	1
SEL	Socioeconomic Level	2.60	1.11	4	1
SEL_IHE	Socioeconomic Level of The Institution of Higher Education	2.41	0.93	4	1

***Note:****S_11 corresponds to the secondary test and S_PRO to the professional test*.

**Table 2:** Description of categorical variables.

Variable	Full Name	Levels	Variable	Full name	Levels
GENDER	Gender	2	DVD	DVD	2
EDU_FATHER	Father's education	12	FRESH	Fresh	2
EDU_MOTHER	Mother's education	12	PHONE	Phone	2
OCC_FATHER	Father's occupation	13	MOBILE	Mobile	2
OCC_MOTHER	Mother's occupation	13	REVENUE	Revenue	3
STRATUM	Stratum	7	JOB	Job	8
SISBEN	Sisben	6	SCHOOL_NAME	School name	3.735
PEOPLE_HOUSE	People in the house	13	SCHOOL_NAT	Nature of School	2
INTERNET	Internet	2	SCHOOL_TYPE	Type of School	4
TV	TV	2	COD_SPRO	Code Saber Pro	12.411
COMPUTER	Computer	2	UNIVERSITY	University	134
WASHING_MCH	Washing machine	2	ACADEMIC_PROGRAM	Academic Program	23
MIC_OVEN	Microwave oven	2	COD_S11	Code Saber 11	12.411
CAR	Car	2			

Table 1 shows the numerical variables of the data set, in the first column they are presented as the variable is coded, the second column the original name of the variable, the third column the general average of the data of that variable, the third column is the deviation of the variable, finally, the fourth and fifth column are the maximum and minimum of each variable correspondingly. Table 2 shows the categorical variables of the data set, the first column is the coded name of the variable, the second column the original name of the variable and finally, the third column represents the levels or categories that each variable possesses. On the other hand, a summary of some variables of the data set for each academic program is presented in Table 3, the first column has the name of the academic program, the second column has the percentage of women belonging to the academic program and the percentage of men is in the third column, in the fourth column the percentage of students who come from a public school and in the fifth column the percentage of students who come from a private school, in the sixth column is the average result of the Engineering Project Formulation variable FEP_PRO and the last column presents the overall average score of the professional evaluation G_SC.

**Table 3:** Summary by engineering program.

Academic Program	% Women	% Men	% Public School	% Private School	FEP_PRO	G_SC
Civil constructions	42.86%	57.14%	85.71%	14.29%	154.36	151.86
Aeronautical Engineering	27.27%	72.73%	43.18%	56.82%	138.52	155.80
Cadastral Engineering and Geodesy	58.97%	41.03%	48.72%	51.28%	78.08	174.60
Civil Engineering	35.87%	64.13%	49.94%	50.06%	144.46	161.11
Control Engineering	41.67%	58.33%	50.00%	50.00%	163.42	177.08
Production Engineering	51.67%	48.33%	46.67%	53.33%	135.32	172.90
Productivity and quality Engineering	55.17%	44.83%	68.97%	31.03%	62.55	162.10
Transportation and road Engineering	48.15%	51.85%	96.30%	3.70%	172.19	167.74
Electric Engineering	21.94%	78.06%	51.44%	48.56%	139.98	173.99
Electromechanical Engineering	14.71%	85.29%	73.53%	26.47%	141.32	148.62
Electronic Engineering	19.55%	80.45%	55.95%	44.05%	145.87	166.87
Electric Engineering and telecommunications	19.15%	80.85%	42.55%	57.45%	149.53	160.43
Industrial Automation Engineering	36.36%	63.64%	68.18%	31.82%	160.41	166.09
Automation Engineering	30.00%	70.00%	20.00%	80.00%	160.30	165.50
Control Engineering	0.00%	100.00%	75.00%	25.00%	65.38	164.75
Control Engineering and industrial automation	0.00%	100.00%	0.00%	100.00%	94.00	113.00
Industrial Engineering	51.97%	48.03%	46.80%	53.20%	147.48	159.04
Mechanical Engineering	12.07%	87.93%	42.64%	57.36%	143.72	166.05
Mechatronics Engineering	7.41%	92.59%	56.79%	43.21%	143.04	151.91
Chemical Engineering	54.50%	45.50%	31.20%	68.80%	152.39	176.70
Textile Engineering	100.00%	0.00%	100.00%	0.00%	190.00	171.00
Topographic Engineering	42.86%	57.14%	50.00%	50.00%	70.74	172.71

## 2 Experimental design, materials, and methods

For the design of the database, the list of crosses that related the code of the secondary test and the code of the professional test of a student was needed, then we proceed to download the databases of both tests according to the years that indicate the codes (Example: SB2006XX crossed with EK2018XX, year 2006 for the secondary test and year 2018 for the professional test of the student). For each database the extraction of the variables of interest is performed, and a filter is applied to the Engineering programs analyzed in the study. Once the filter is finished, the two databases are joined in a documented document through the crossings, followed by this, the data is encoded and cleaned in the desired format. The format was carried out in such a way that it would facilitate to identify the information flow of the results of the secondary and professional test; It also allowed an easy interpretation and manipulation of them. The data was manipulated with the tidyr library [1] and dyplr [2] of the R software [3].

## Appendix Supplementary materials

Image, application 1
